# Orchestrating an immune response to cancer with cellular reprogramming

**DOI:** 10.1038/s41435-023-00237-4

**Published:** 2023-12-15

**Authors:** Olga Zimmermannova, Alexandra Gabriela Ferreira, Carlos-Filipe Pereira

**Affiliations:** 1 Molecular Medicine and Gene Therapy, Lund Stem Cell Center, Lund University, Lund, Sweden; 2 Wallenberg Center for Molecular Medicine at Lund University, Lund, Sweden; 3 CNC-Centre for Neuroscience and Cell Biology, University of Coimbra, Coimbra, Portugal

**Keywords:** Antigen-presenting cells, Immunotherapy, Immune evasion

In recent years, immunotherapy has transformed cancer treatment by harnessing the patient’s own immune system. However, cancer cells develop mechanisms to evade immune surveillance, compromising the body’s natural defences against cancer. The lack of immune control and immunotherapy failure can be attributed to both tumour intrinsic and extrinsic factors favouring the survival of tumour cells. These encompass primarily the downregulation of antigen presentation and major immunohistocompatibility complex (MHC) molecules on the cell surface, increasing tumour heterogeneity and exclusion or functional impairment of immune effectors within the tumour microenvironment (TME) [1]. Recent studies have emphasised that the recruitment and activation of T cells and effective responses to immunotherapy are orchestrated by antigen-presenting type 1 conventional dendritic cells (cDC1). However, in cancer patients, the cDC1 subset is frequently depleted or functionally impaired, hindering the immune system’s capacity to recognise and target cancer cells [2]. Nonetheless, because cDC1 play an essential role in initiating multiple layers of anticancer immunity, they represent a promising target for therapeutic interventions aimed at counteracting tumour immune evasion mechanisms. The challenge of the field has been to find a therapeutic source of cDC1s. Given their low abundance, cDC1 cannot be obtained from the peripheral blood of patients or donors. Moreover, differentiation protocols using blood monocytes do not yield a pure cDC1 population but rather a mix of DC subsets with conflicting functions and limited ability to cross-present antigens [2]. We were inspired by the potential of cell fate reprogramming for cell replacement therapy and identified a combination of three transcription factors—PU.1, IRF8, and BATF3 (collectively termed as PIB)—that control cDC1 cellular identity and are capable of reprogramming mouse and human fibroblasts into professional antigen-presenting cells that closely resemble natural cDC1 cells in terms of morphology, phenotype, transcriptome and function [3]. We further elucidated mechanistic aspects by mapping transcription factor chromatin engagement at the early stages of reprogramming and showed that PU.1 govern the cooperative binding of IRF8 and BATF3 to silence fibroblast genes and activate the cDC1 transcriptional programme [4].

**Fig. 1:**
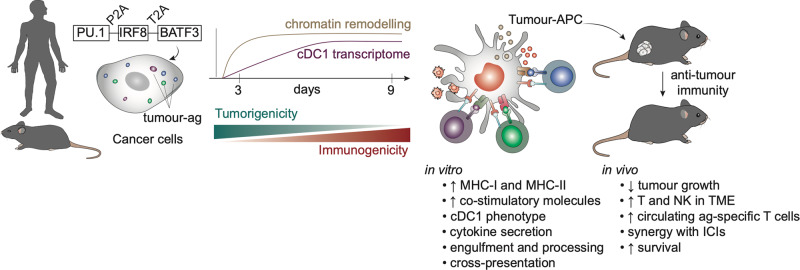
Restoring antitumor immunity through a cDC1 reprogramming strategy. Mouse and human cancer cells were shown to be reprogrammed into tumour-antigen-presenting cells (APCs) through overexpression of the transcription factors PU.1, IRF8, and BATF3. The reprogramming process was fast, with global chromatin remodelling occurring in the first 3 days, followed by a stepwise adoption of type 1 conventional dendritic cell (cDC1) transcriptome until day 9. Tumour-APCs lose tumorigenicity and upregulate antigen presentation machinery (MHC-I, MHC-II and costimulatory molecules) that enhance intrinsic tumour immunogenicity. Notably, tumour-APCs acquired cDC1 functional features including ability to uptake dead cells, inflammatory cytokine secretion, and processing and presentation of endogenous and exogenous antigens (ag) to CD8+ T cells. In vivo, intratumoral administration of tumour-APCs delays tumour growth, promotes T and NK cell infiltration in the tumour microenvironment (TME), and synergises with immune checkpoint inhibitors (ICI) to prolong survival.

Considering the critical role of cDC1 in presenting cancer antigens for effective anti-tumour immunity, we hypothesised that inducing cDC1 identity and antigen presentation directly in cancer cells could restore tumour immunogenicity and counteract key tumour immune evasion mechanisms. Through enforced expression of PIB, we demonstrated that a large array of human cancer cell lines could be converted into highly immunogenic tumour-antigen-presenting cells (tumour-APCs). These reprogrammed tumour-APCs exhibited similarities to natural cDC1s at the epigenetic and transcriptomic levels, expressed costimulatory molecules on the cell surface, and adopted functional features of cDC1s. Importantly, immunopeptidomic analysis confirmed that tumour-APCs restored the presentation of tumour-antigens, leading to increased immunogenicity and active killing of reprogrammed cells by T cells specific against endogenous cancer antigens. Furthermore, tumour-APCs responded to Toll-like-receptor stimuli and secreted pro-inflammatory cytokines, engulfed dead cells, and processed and presented both endogenous and internalised tumour antigens, leading to the productive activation of T cells. In addition to the acquired antigen-presenting capacity, tumour-APCs reduced cancer cell tumorigenic capabilities in vitro and in vivo. cDC1 reprogramming imposed a tumour-APC signature over a large spectrum of primary patient cancer tissues. To validate that cancer transformation does not limit reprogramming, we showed that tumour-APCs were generated at comparable efficiencies and fidelity at single cell level both from aneuploid (cancerous) and diploid cells present in the majority of patient tumour tissues. Notably, reprogrammed primary melanoma activated naïve antigen-specific T cells, and elicited cytotoxic killing responses from tumour infiltrating lymphocytes from the same patient. In vivo, administration of tumour-APCs into established melanoma tumours in mice notably delayed tumour growth and induced regression in low immunogenic syngeneic models. The induced anti-tumour immunity was particularly striking when combined with immune checkpoint inhibitors αPD-1 and αCTLA-4, leading to complete tumour regressions [5]. In parallel, another recent study demonstrated that the macrophage reprogramming factors PU.1 and CEBPα could also induce an immune response in leukaemia [6]. This capacity to restore anti-tumour immunity and drive cancer eradication underscores the potential of direct cellular reprogramming for cancer immunotherapy (Fig. 1) [5, 6].

In contrast to prior cancer reprogramming efforts, cDC1 reprogramming aimed not only to reduce malignancy but also to create cancer cells that actively present cancer antigens. The generation of tumour-APCs offers an efficient and broadly applicable ‘one-fits-all’ reprogramming system. However, moving towards clinical translation presents questions to address, particularly in understanding the factors that determine reprogramming success. In our study, we induced tumour-APC populations in more than 60 cancer cell lines and primary cancer cells. We observed significant variability in the reprogramming efficiency and a proportion of cancer cells responded only by generating partially reprogrammed cells. While we have shown that partially reprogrammed cells also display reduced tumorigenicity and acquire cDC1 functional features, it indicates the existence of genetic or epigenetic barriers hindering the completion of the process. These barriers differ from those encountered in pluripotency reprogramming, such as the growth rate or TP53 mutations [2, 5]. Therefore, identifying barriers and facilitators of cDC1 reprogramming and the development of spheroid-based platforms to predict the efficacy of tumour-APC-based immunotherapy may facilitate the selection of patients who will benefit the most from this therapy.

In our study, tumour-APC intratumoral administration resulted in tumour regression and expansion of tumour antigen-specific T cells. While the presence of T cell clones targeting multiple cancer antigens in peripheral blood and lymph nodes suggested systemic immune responses, it remains to be experimentally validated whether tumour-APCs induce systemic immunity and durable immunological memory in syngeneic tumour models. The ability of tumour-APCs to deliver abscopal effect and target distant metastases is particularly important, given that immunotherapy is often considered for recurrent and advanced cancer cases. The establishment of immunological memory is vital to prevent cancer relapse that would otherwise significantly limit patients’ survival.

An important question is how to translate cDC1 reprogramming of cancer cells into an immunotherapy. Autologous-based therapy based on ex vivo reprogramming of patients’ cancer cells can be an option for malignancies such as leukaemia that do not form localised tumours [6]. However, this approach comes with logistic and manufacturing challenges as well as high associated costs. On the other hand, in vivo reprogramming via intratumoral delivery of reprogramming factors may offer both immunity against patient-specific cancer antigens and the convenience of an off-the-shelf therapy. Tumour-APC-based gene therapy may be an effective solution, allowing large-scale manufacturing at lower costs, thereby making cDC1 reprogramming a personalised yet readily available treatment solution. As we learnt from regenerative medicine and the reprogramming of retina or pancreatic β cells, reprogrammed cells in vivo reach a more mature state and show improved functionality and reprogramming fidelity, better resembling their naturally occurring counterparts. However, in the context of cancer, in situ reprogramming brings TME as a significant factor that may impact reprogramming. Given the profound impact of TME on cell behaviour and proliferation, it will be important to assess the effect of tumour-associated immune suppression, together with hypoxia and other local factors, on the reprogramming process and its efficacy. Furthermore, the development of an optimal delivery platform for reprogramming factors is another critical aspect of entering clinical trials and first-in-human studies.

In conclusion, immune evasion mechanisms such as inhibition of antigen presentation have long been considered major mechanisms allowing cancer cells to disappear from the immune radar. Our cell fate reprogramming-based approach presents an exciting opportunity to counteract these immune evasion mechanisms by addressing tumour heterogeneity, loss of antigen presentation, combined with the replenishment of functional cDC1s within the TME. Our study establishes the foundations for the development of a novel cancer immunotherapy modality that allows in situ reprogramming of cancer cells into tumour-antigen-presenting cells, bridging cell fate reprogramming and immunotherapy.
